# Synergistic Antifungal Activity of Graphene Oxide and Fungicides against Fusarium Head Blight In Vitro and In Vivo

**DOI:** 10.3390/nano11092393

**Published:** 2021-09-14

**Authors:** Xiuping Wang, Fei Peng, Caihong Cheng, Lina Chen, Xuejuan Shi, Xiaoduo Gao, Jun Li

**Affiliations:** 1 Analysis and Testing Center, Hebei Normal University of Science and Technology, Qinhuangdao 066000, China; wangxiuping0721@163.com (X.W.); flyer5212528@163.com (F.P.); cch20059@126.com (C.C.); 2 Hebei Key Laboratory of Active Components and Functions in Natural Products (under Planning), Hebei Normal University of Science and Technology, Qinhuangdao 066004, China; 3 College of Agronomy and Biotechnology, Hebei Normal University of Science and Technology, Qinhuangdao 066000, China; chen830212@163.com (L.C.); xuejuanshi@163.com (X.S.); gaoxiaoduo30503@163.com (X.G.); 4 Oil Crops Research Institute, Chinese Academy of Agricultural Sciences, Wuhan 430070, China

**Keywords:** graphene oxide, fungicides, synergistic antifungal activity, nanofungicides, plant pathogenic fungi

## Abstract

Plant pathogens constantly develop resistance to antimicrobial agents, and this poses great challenges to plant protection. Therefore, there is a pressing need to search for new antimicrobials. The combined use of antimicrobial agents with different antifungal mechanisms has been recognized as a promising approach to manage plant diseases. Graphene oxide (GO) is a newly emerging and highly promising antimicrobial agent against various plant pathogens in agricultural science. In this study, the inhibitory activity of GO combined with fungicides (Mancozeb, Cyproconazol and Difenoconazole) against *Fusarium graminearum* was investigated in vivo and in vitro. The results revealed that the combination of GO and fungicides has significant synergistic inhibitory effects on the mycelial growth, mycelial biomass and spore germination of *F. graminearum* relative to single fungicides. The magnitude of synergy was found to depend on the ratio of GO and fungicide in the composite. In field tests, GO–fungicides could significantly reduce the disease incidence and disease severity, exhibiting a significantly improved control efficacy on *F. graminearum*. The strong synergistic activity of GO with existing fungicides demonstrates the great application potential of GO in pest management.

## 1. Introduction

Graphene oxide (GO) is composed of sp2-hybridized carbon atoms hexagonally arranged in single-atom thickness [1]. The special electronic, mechanic and optical characteristics of graphene structure suggest that GO may be a promising next-generation antimicrobial material [2,3]. Recent studies have revealed that GO has a broad spectrum of bactericidal activity, and can significantly inhibit the growth of both Gram-negative and Gram-positive bacteria [4,5]. As a newly emerging antimicrobial agent against plant pathogenic bacteria and fungi, GO has attracted great research interests and is believed to have great application potential in agricultural production [6,7,8,9]. It has been demonstrated that GO can inactivate a multitude of bacteria, such as copper-resistant *Ralstonia solanacearum* [7], *Xanthomonas oryzae* pv. *oryzae*, *Pseudomonas syringae* and *Xanthomonas campestris* pv. *undulosa* [8]. In addition, GO can strongly inhibit the mycelial growth and spore germination of various plant fungal pathogens, such as *Fusarium graminearum*, *Fusarium poaea* and *Fusarium* *oxysporum* [9]. The high antibacterial activity of GO against plant pathogens makes it a promising candidate for the control of phytopathogens.

Plant diseases have caused severe economic losses ever since the beginning of agriculture [10]. It has been estimated that about 85% of plant diseases are caused by fungi and bacteria in nature. To combat fungi and bacteria, various types of chemical fungicides have been developed and applied, owing to their high efficacy against causal agents of the disease and low cost [11]. However, under the long-term application of chemical fungicides in agriculture, microorganisms have gradually developed antimicrobial resistance, which largely reduces the effectiveness of fungicides [12]. In this case, fungicides are excessively and repeatedly used to control the diseases, causing a series of problems such as chemical residue, environmental pollution and some undesirable effects on non–target organisms [13,14,15]. Thus, the development of alternative strategies to improve the efficacy of fungicides and, at the same time, minimize their side effects has been an important research topic in both agricultural and environmental chemistry.

Recently, the application of nanomaterials to the plant disease protection has become a new research hotspot [16]. It has been proposed that the use of nanomaterials for nano-based smart formulation of fungicides (termed as nanofungicides) may reduce the use of active ingredients and harmful chemicals to non-target organisms, thus limiting the development of pathogen resistance and adverse effects on the environment [17,18,19]. However, the application of nanomaterials in agriculture, particularly for plant protection and production, is still at the initial stage of development in the academic community.

For practical application, in the present work, we combined existing fungicides with the nanomaterial GO to formulate new GO–fungicides against the plant pathogenic fungus *Fusarium graminearum*, a prevalent and aggressive pathogen causing Fusarium head blight (FHB) in cereal crops worldwide [20]. Mancozeb (Man) is a very important protective contact dithiocarbamates fungicide. Cyproconazol (Cyp) and Difenoconazole (Dif) are systemic triazole fungicides, and have preventive and therapeutic effects. These three fungicides have broad-spectrum antifungal activities and a wide range of applications. However, the extensive use of these fungicides in plant disease protection has led to the emergence of fungicide resistance, which largely reduces the effectiveness of fungicides. Thus, Man, Cyp and Dif were chosen as model fungicides in this study. The aim of this study was to explore the potential synergistic effect of GO when combined with fungicides to improve the efficacy of disease control. The synergistic effects of GO on the antifungal activity of Man, Cyp and Dif against the *F. graminearum* were evaluated in vitro and in vivo. The results demonstrated that the combination of GO with fungicides can contribute to highly potent antifungal activity. To our knowledge, this is the first report of the synergistic antifungal effect between fungicides and GO, which may provide important implications for the better design of graphene–based fungicides or the application of other fungicides.

## 2. Materials and Methods

### 2.1. Materials

All chemicals were of analytical grade and used as received, without further purification. Graphite was purchased from Qingdao Tianhe Graphite Co. Ltd. (Qingdao, Shandong province, China), with an average particle diameter of 4 mm (99.95% purity). All other reagents were obtained from the Tianjin No. 3 Chemical Plant. Dif (analytical standard grade) (Tianjin, China), Cyp (ACS grade) and Man (ACS grade) were purchased from Sigma–Aldrich (Shanghai, China).

### 2.2. Preparation and Characterization of GO

Commercially available graphite powder was oxidized and exfoliated to GO by a modified Hummers method [21]. The morphology of GO and GO–fungicide nanocomposites was measured by transmission electron microscope (TEM, Hitachi H-7650, Tokyo, Japan).

### 2.3. Preparation of GO–Fungicide Nanocomposites

GO–fungicide nanocomposites were prepared by physical loading of fungicides onto the surface of GO [22]. Briefly, fungicides of different mass were dispersed in 2 mL of acetone and Tween 80 solution (1:1, v/v), and then GO was added to make the final mass ratio of GO and fungicide to be 1:9, 2:8, 3:7, 6:4, 5:5, 4:6, 7:3, 8:2 and 9:1. The mixture was then stirred in the dark for 24 h. The obtained product was washed with acetone and tween 80 solution (AT).

### 2.4. Fungus

*F. graminearum* strains (PH-1) were provided by the State Key Laboratory of Agricultural Microbiology of Huazhong Agricultural University (Wuhan, Hubei Province, China). Fungal cultures were maintained on a potato dextrose agar (PDA) slant at 4 °C. The old cultures were moved to a fresh slant every two months, so as to avoid the decline of strain viability.

### 2.5. Bioassay of Antifungal Activity of GO–Fungicide Nanocomposites In Vitro by Mycelial Growth Test

The antifungal effects of Man, Cyp and Dif individually or in combination with GO on the mycelial growth and biomass of *F. graminearum* were analyzed, as presented in Table 1. Preliminary investigations showed that the inhibition rate of the three fungicides against the mycelial growth of *F. graminearum* could reach 10–90% at different concentrations. Thus, different concentrations of the three fungicides (presented in Table 1) were chosen for further bioassay testing. Briefly, *F. graminearum* was inoculated onto solid PDA (Nantong Kaiheng Biotechnology Development Co., Ltd, Nantong, (Jiangsu Province, China) (for mycelial growth test) and PD medium (without agar, for biomass test) containing different concentrations of GO, fungicides and GO–fungicides. An equal volume of AT solution without any fungicides was processed similarly as the control. After 120 h of incubation at 24 ± 2 °C, the mycelial growth and mycelial biomass of *F. graminearum* in each treatment were observed. The inhibition rate of mycelial growth (I, %) was calculated as follows:

I = (1 − Dt/Dc) × 100%,

where Dc is the mycelial diameter or biomass measured in the control set, and Dt represents the mycelial diameter or biomass measured in the treatment sets after 120 h of incubation [23]. The antifungal effect was measured under a totally random design with four replications. The mycelial dry weight was measured after repeated washing of the mycelial pellets with distilled water and drying at 50 °C, to a constant weight.

### 2.6. Bioassay of Antifungal Activity of GO–Fungicide Nanocomposites In Vitro by Spore Germination Test

For spore germination tests, the spores of *F. graminearum* were prepared as previously described [24]. Spores were first incubated in 3% green-bean-soup liquid medium for 5 days, and then collected by centrifugation at 4000 rpm for 5 min and washed with sterile distilled water three times. The concentration of the spore suspensions was first adjusted to 1 × 10^6^ spores mL^−1^, followed by the mixing of the same volume of spore suspension with GO, fungicides and GO–fungicides in the tube to achieve the final concentrations presented in Table 1. The control sample was prepared by mixing the same volume of spore suspension and AT. About 30 μL of mixture containing different concentrations of GO, fungicides and GO–fungicides was transferred to a concave slide and further incubated at 28 °C for 5 h, in complete darkness. Five concave slides were performed for each treatment, and the mean values were compared. Micrographs were taken by a digital camera (Leica, Wetzlar, Germany) linked to a Leica microscope (Leica, Wetzlar, Germany). 

Spore germination and mycelial growth inhibition of the treatments were calculated in percentage with the equation as follows:

*I*_R_ = [(*C* − *T*)/*C*] × 100,

where *I*_R_ is the inhibition rate of spore germination, *C* is the number of germinated spores of the control and *T* is the number of germinated spores of the treatment groups. 

### 2.7. Field Trials

Field trials were carried out in the Experimental Station of Hebei Normal University of Science and Technology. The wheat cultivar ‘LunXuan 103’, which is widely planted in Hebei, China, was used in this experiment. The antifungal activity of GO–fungicides was measured with single floret injection, as previously described [25]. During the anthesis period, point inoculation (10 μL mixture of spore suspension with GO, fungicide and GO–fungicide suspensions, v:v = 1:1) was performed in the central spikelet of selected spikes. Small plastic bags were used to cover the inoculated spikes for three days to maintain humidity for the spike in order to facilitate disease development. Spikes inoculated with AT in sterile water served as the negative control. In total, 100 spikes (25 on each subplot, four replicates) were evaluated. Scoring of diseased spikelets in each inoculated spike was performed at 7 d post-inoculation. Quantitative symptoms of infection, such as disease incidence (DI, the percentage of spikelets with symptoms in the total number of spikelets) and disease index (DS), were visually evaluated independently at 7 d after inoculation by using a 0–100% severity scale previously described [26].

### 2.8. Structural and Morphological Characterization by SEM and TEM

The morphological changes of *F. graminearum* were further investigated by scanning electron microscope (SEM, JEOL JSM–6700F) (JEOL, Tokyo, Japan,) and TEM (Hitachi H–7650) (Hitach, Tokyo, ) after treatment with different concentrations of GO, fungicides and GO–fungicides. Fungal mycelia obtained from PD medium were fixed with osmium tetroxide (O_s_O_4_), overnight, at 4 °C; dehydrated in ethanol; air-dried; sputter-coated with chromium for 5 min; and mounted on copper grids. The samples were further investigated by SEM and TEM examination [23].

### 2.9. Data Analysis

Data from the bioassays were subjected to analysis of variance (ANOVA). The Duncan multiple comparison test was used to determine the significant difference among treatments (*p* > 0.05). The 50% effective concentration (EC_50_) values were calculated by regressing the percentage growth inhibition against the log-transformed fungicide concentration. Each treatment was performed with four replicates, and the experiment was repeated for four times. The SR (synergism ratio) was calculated as follows:

SR = EC_50_ (i)/EC_50_ (m)

where EC_50_ (i) is the observed EC_50_ value of individual fungicides, while EC_50_ (m) is the observed EC_50_ value of GO–fungicides. An SR value below 0.5 indicates antagonistic interactions between fungicides in the mixture, an SR value between 0.5 and 1.5 represents additive interactions and an SR value higher than 1.5 indicates synergistic interactions [27].

## 3. Results and Discussion

### 3.1. Screening of the Optimal Ratio of GO and Fungicides

**Figure 1:**
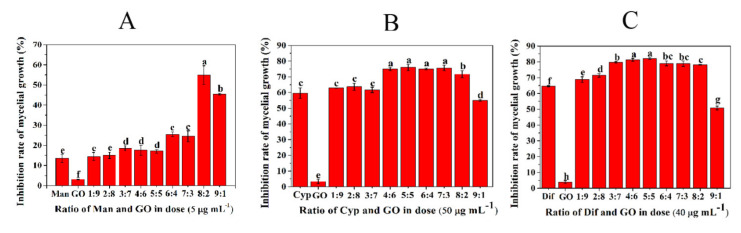
Inhibitory activities of single fungicides or in combination with GO at different mass ratios against mycelial growth of *F. graminearum*. Inhibitory activities of Man and GO (**A**), Cyp and GO (**B**) and Dif and GO (**C**) at different mass ratios against mycelial growth of *F. graminearum*. Data are mean ± stand error (SE). Error bars represent the SE (N = 4). Different lowercase letters indicate significant differences between treatments (*p* < 0.05).

In order to screen the optimal combination ratio of GO with three different fungicides, the inhibitory effect of GO–fungicides combined at different ratios on mycelial growth was tested. As shown in Figure 1A, the inhibition rate of single Man and GO was 14.5% and 3.1%, respectively, while the inhibition rate of Man–GO combined at 8:2 was 54.7%. Hence, Man–GO combined at 8:2 was screened as the optimal ratio. The Cyp–GO combined at the ratios from 4:6 to 7:3 showed higher antifungal activities than other combinations (Figure 1B). The Dif–GO combination at the ratios of 4:6 and 5:5 resulted in the highest antifungal activity (Figure 1C). Considering the cost and feasibility of practical application of GO–fungicides, we chose Man–GO at 8:2 and Cyp–GO and Dif–GO at 5:5 as the best combinations for subsequent characterization and antifungal activity analysis. These results confirmed the findings of Yang et al., who claimed that the antifungal activity of two and more fungicides can be synergized only when these fungicides are combined with the appropriate ratio [28]. Moreover, it has been reported that the dispersibility of nanomaterials affects their antimicrobial effects [29]. Thus, the dispersibility of GO and GO–fungicides combined at different ratios was investigated by TEM.

### 3.2. Characterization of Formulated GO–Fungicides

**Figure 2:**
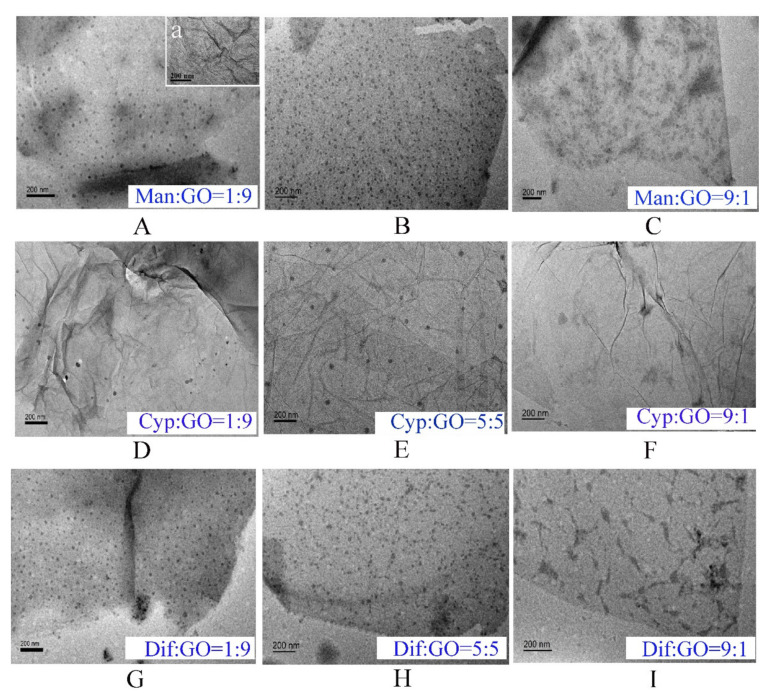
Characterization of the formulated GO–fungicides. TEM image of GO (Aa); GO–fungicides with the ratio of Man and GO at 1:9 (**A**), 8:2 (**B**) and 9:1 (**C**); the ratio of Cyp and GO at 1:9 (**D**), 5:5 (**E**) and 9:1 (**F**); and the ratio of Dif and GO at 1:9 (**G**), 5:5 (**H**) and 9:1 (**I**), respectively.

The surface of GO has been demonstrated to be generally favorable for the adsorption of molecules with low water solubility through electrostatic attraction, hydrophobic interaction and π−π stacking [30,31]. In general, most active ingredients of fungicides are insoluble in water, which can explain the result that numerous insoluble ingredients of Man, Cyp or Dif were adsorbed on GO (Figure 2). As shown in Figure 2A(a), the GO sheets are thin and smooth with small wrinkles. However, the surfaces of Man–GO (Figure 2A–C), Cyp–GO (Figure 2D–F) and Dif–GO (Figure 2G–I) have some black spots, which were not observed on the surface of GO. The black spots formed on the GO surface showed different shapes when GO was combined with different fungicides, indicating that the appearance of these black spots was due to the adsorption of Man, Cyp and Dif on GO sheets. A comparison of different ratios in Figure 2A–C shows that at the Man-to-GO mass ratio of 1:9, Man could be adsorbed on GO surface with better dispersibility, but the adsorption amount was relatively small (Figure 2A). At the Man-to-GO mass ratio of 9:1, the Man adsorbed on GO surface showed a tendency of aggregation (Figure 2C). Figure 2B shows that, at the Man-to-GO mass ratio of 8:2, a larger amount of Man could be adsorbed on the surface of GO with a better dispersibility. Similar phenomena were observed for Cyp–GO (Figure 2D–F) and Dif–GO (Figure 2G–I). The aggregation of Man at the ratio of 9:1 may be ascribed to the enhancement of hydrogen bonds between molecules of Man, while the aggregation of Cyp and Dif adsorbed on GO surface may be due to the stronger π−π stacking effect between the aromatic rings of Cyp or Dif than that between Cyp or Dif and GO. These results indicate that the Man, Cyp and Dif adsorbed on GO surface have the highest adsorption capacity and the best dispersibility at the combined ratio of 8:2, 5:5 and 5:5, respectively, which may be the reason for the better antifungal activity of GO–fungicides at these ratios. Thus, we carried out the following bioassay test with the abovementioned optimal ratios.

### 3.3. Synergistic Inhibitory Activity of GO–Fungicides on the Mycelial Growth of F. graminearum

**Figure 3:**
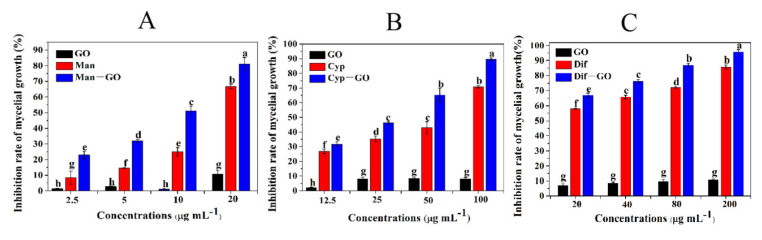
Inhibitory activities of GO, fungicides and GO–fungicides on the mycelial growth of *F. graminearum*. The inhibition rate of mycelial growth was tested at different concentrations of GO and Man–GO (**A**), Cyp–GO (**B**) and Dif–GO (**C**) for 120 h at 24 ± 2 °C. Data are mean ± SE. Error bars represent the SE (N = 4). Different lowercase letters indicate significant differences between treatments (*p* < 0.05).

Mycelia are infection structures that invade plant tissues and the vascular system to cause systemic plant infection. Figure 3 shows the inhibitory effects of four concentrations of GO and each fungicide and GO–fungicide on the growth of mycelia relative to the control. All fungicides and GO–fungicides exhibited certain inhibitory effects on the mycelial growth in a dose-dependent manner. Figure 3A shows that GO could only achieve a 1.56–9.21% inhibition rate on mycelial growth at the concentrations of 2.5–20 μg mL^−^^1^. Man treatments at different concentrations could achieve an inhibition rate of 9.87–67.4% on mycelial growth. However, Man–GO composite showed a much higher efficacy in controlling the mycelial growth of *F. graminearum* relative to GO and Man alone. Man–GO composite could inhibit the mycelial growth by nearly 25% at the lowest concentration (2.5 μg mL^−^^1^), and even could achieve an inhibition rate of 80% at the highest concentration (20 μg mL^−^^1^), demonstrating that Man–GO composite has a superior antifungal effect than Man and GO alone.

Further, the synergistic inhibitory effect of GO and Cyp on the mycelial growth was also investigated. Figure 3B shows that GO treatments could only inhibit the mycelial growth by less than 10% at any tested concentrations, and the inhibition rate of mycelial growth by Cyp treatments with various concentrations was merely 28.91–70.04%. However, Cyp–GO composite could inhibit the mycelial growth by 34.62–89.13%, indicating that the incorporation of GO can significantly increase the toxicity of Cyp to the fungi.

Similarly, we also investigated the synergistic inhibitory effect of GO and Dif on the mycelial growth. The inhibition rate of Dif treatments at various concentrations on mycelial growth ranged from 55.61% to 90.64%; in contrast, the Dif–GO composite at different concentrations could inhibit the mycelial growth by 80.82–95.72% (Figure 3C), suggesting that the incorporation of GO can improve the antifungal performance of Dif. 

### 3.4. Synergistic Inhibitory Activities of GO–Fungicides on the Mycelial Biomass of F. graminearum

**Figure 4:**
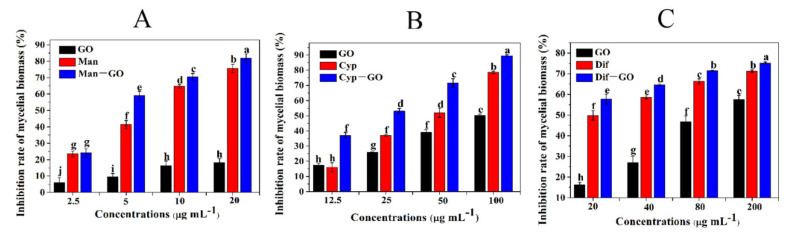
Inhibitory activities of GO, fungicides and GO–fungicides on the mycelial biomass of *F. graminearum*. The inhibition rate of mycelial biomass was tested at different concentrations of GO and Man–GO (**A**), Cyp–GO (**B**) and Dif–GO (**C**) for 120 h at 24 ± 2 °C. Error bars represent the standard error (N = 4). Different lowercase letters indicate significant differences between treatments (*p* < 0.05).

The synergistic inhibitory activity of GO–fungicides on the mycelial biomass of *F. graminearum* was investigated. As shown in Figure 4, all GO, fungicides and GO–fungicides could inhibit the biomass of *F. graminearum* in a dose-dependent manner. Figure 4A shows that the inhibition rate of GO treatments on the mycelial biomass was 4.60–18.26% at different tested concentrations, and that of Man treatments was 23.72–71.21%, respectively. However, Man–GO composite showed a much higher efficacy in controlling the mycelial biomass of *F. graminearum*, with inhibition rates of 24.57–82.23% at different concentrations.

Similarly, when GO was combined with Cyp and Dif, the toxicity of the resulting Cyp–GO (Figure 4B) and Dif–GO (Figure 4C) composites was significantly increased when compared with that of GO and fungicide alone. These results suggest that GO has a superior synergistic effect to promote the toxicity of Man, Cyp and Dif against the mycelial biomass of *F. graminearum*.

### 3.5. Synergistic Inhibitory Activity of GO–Fungicides on the Spore Germination of F. graminearum

**Figure 5:**
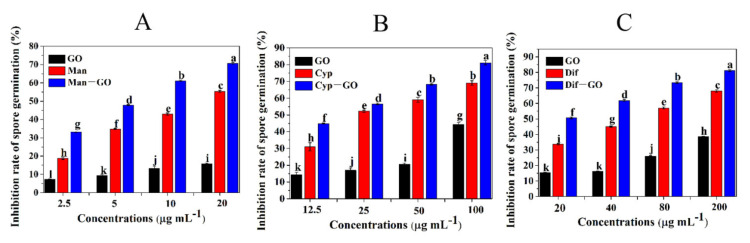
Inhibitory activities of GO and GO–fungicides on the spore germination of *F. graminearum*. The inhibition rate of spore germination was evaluated at different concentrations of GO and Man–GO (**A**), Cyp–GO (**B**) and Dif–GO (**C**) after 5 h of incubation. Error bars represent the standard error (N = 5). Different lowercase letters indicate significant differences between treatments (*p* < 0.05).

Spore germination is a critical developmental stage for all filamentous fungi in the life cycle, as well as represents a preliminary step for the development of tools to be further used for characterizing the early interaction events between pathogens and hosts [32]. To gain more insights into the functions of GO–fungicides, we investigated the antifungal activities of GO, fungicides and GO–fungicides against the spore germination of *F. graminearum*. Figure 5 shows the inhibition rates of GO, fungicides and GO–fungicides on *F. graminearum* spore germination at different doses. Figure 5A shows that the inhibition rate of single GO and Man on spore germination was 7.39–15.86% and 18.80–55.38%, respectively, while their combination (Man–GO composite) resulted in an inhibition rate of 33.24–70.67%, which is significantly higher than that of either Man or GO treatment alone. Similarly, Figure 5B, C respectively reveals that the Cyp–GO and Dif–GO composites had higher inhibitory activities on spore germination than Cyp and Dif alone. These results demonstrate that GO has a significant synergistic antifungal activity when combined with Man, Cyp and Dif.

Table 2 shows the EC_50_ values of single fungicides and GO–fungicide composites against the mycelial growth, mycelial biomass and spore germination of *F. graminearum*. The observed effects were expressed as EC_50_ values derived from a probit analysis with a 95% confidence limit. As shown in Table 2, the EC_50_ value of single Man was 15.70 µg mL^−1^, while that of Man–GO composite was 7.80 µg mL^−1^ for mycelial growth, with a synergism ratio (SR) of 2.01, indicating that Man–GO composite was about 2.10-fold more potent than single Man against mycelial growth. The EC_50_ value of single Cyp and Dif was 40.65 and 5.8 µg mL^−1^, respectively, while that of Cyp–GO and Dif–GO composites was 27.05 and 2.33 µg mL^−1^, respectively, indicating that the supplementation of GO could significantly decrease the EC_50_ value. The SR for the EC_50_ values of Cyp–GO and Dif–GO composites was 1.50 and 2.49, respectively, indicating a 1.50- and 2.49-fold increase in antifungal activity against the mycelial growth relative to that of single Cyp and Dif, respectively.

The EC_50_ values of single fungicides and GO–fungicides against the mycelial biomass of *F. graminearum* were shown in Table 2. The EC_50_ value of single Man was 6.61 µg mL^−1^, while that of GO–Man composite was 4.09 µg mL^−1^, with an SR of 1.61, indicating that Man–GO composite is 1.61-fold more potent than single Man in inhibiting the mycelial biomass of *F. graminearum*. The EC_50_ value of Cyp and Dif was 40.94 and 17.83 µg mL^−1^, respectively, while that of Cyp–GO and Dif–GO composites was 21.03 and 7.12 µg mL^−1^, respectively, indicating that the incorporation of GO could significantly decrease the EC_50_ value. The SR for the EC_50_ values of Cyp–GO and Dif–GO composites was 1.95 and 2.50, respectively, indicating a 1.95-fold and 2.50-fold increase in antifungal activity against the mycelial biomass relative to that of single Cyp and Dif, respectively. 

Similarly, the EC_50_ values of single Man, Cyp and Dif were 14.20, 30.19 and 56.03 µg mL^-1^ against the spore germination of *F. graminearum*, while those of Man–GO, Cyp–GO and Dif–GO composites was 5.90, 17.16 and 18.04 µg mL^−1^, respectively. The SR for the EC_50_ values of Man–GO, Cyp–GO and Dif–GO composites was 2.40, 1.76 and 3.10, indicating a 2.40-, 1.76- and 3.10-fold increase in antifungal activity against the spore germination relative to that of single Cyp and Dif, respectively.

### 3.6. Control Efficiencies of GO and GO–Fungicides on F. graminearum in the Field

Table 3 shows that the GO–fungicides could significantly reduce the DI and DS of FHB in the field. Application of GO–fungicides significantly decreased the DI in comparison with the TW in sterile water (Table 3). The Man–GO, Cyp–GO and Dif–GO composites showed significant control efficiencies of 61.19%, 75.26% and 50.99% (*p* < 0.05), respectively, indicating that these GO–fungicides have stronger antifungal activities than single fungicides, and GO can be considered as a highly promising synergist for fungicides in plant protection. It was reported that the control efficacy of phenamacril and Carbendazim at 250 µg mL^−1^ against *F. graminearum* was 70.21% and 71.47%, which is higher than Man–GO and Dif–GO, while lower than that of Cyp–GO [33]. This phenomenon may be due to the differences in fungal strains used in the test and the experimental methods.

In this study, GO exhibited a low antifungal activity against the mycelial growth of *F. graminearum* at low concentrations, and the antifungal activity did not exceed 10% even at a concentration as high as 200 µg mL^−1^. However, when combined with fungicides, the antifungal activity of GO against the mycelial growth of *F. graminearum* was significantly increased. Moreover, GO was also observed to have synergistic effects to promote the toxicity of Man, Cyp and Dif against the mycelial biomass and spore germination of *F. graminearum*. We speculate that the enhancement of antifungal activity by the incorporation of GO may be ascribed to the context of GO–fungicide composites, which can enhance the effects of fungicides by improving the dispersibility and thus increasing the contact between the fungicide and fungi. GO has good water dispersibility, but fungicides are generally not dispersible in water. The adsorption of fungicide on GO after combination at the optimal ratio can improve the water dispersibility of the fungicide. In addition, the dispersibility of materials may strongly affect their interactions with organisms [34]. Materials with higher dispersibility, such as GO–fungicides, may have higher antifungal activity than those with lower dispersibility (such as Man, Cyf and Dif), possibly due to more opportunities for the fungicides to interact with the fungi. Similar results have been obtained in other nanomaterials. For instance, GO can form stable dispersions with small nanosheets, and therefore exhibits higher toxicity to various bacterial cells and fungi than reduced graphene oxide aggregates [9,22]. Similarly, functionalized carbon nanotubes have stronger toxicity than non-functionalized CNT aggregates [35]. Therefore, it can be speculated that GO improves the antifungal activity of fungicides mainly by enhancing their dispersibility.

Additionally, in the process of pesticide formulation, most of the pesticide active ingredients are hydrophobic compounds, and it can be processed into pesticide formulation only with the help of organic solvents, such as benzene and toluene [36]. The usage of large amount of organic solvents to dissolve hydrophobic active ingredients in pesticide formulations greatly increases the pollution of pesticides to the environment and the toxicity of non-target organisms. GO is constituted by a single-atom-thick lattice of honeycomb-like sp2 bonded carbon atoms and includes abundant oxygen-containing polar functionalities, such as carbonyl, epoxide, carboxyl and hydroxyl groups [37]. Due to the abundant oxygen functionalities, GO can be easily dispersed in organic solvents, water and different matrices [38]. The application of GO as water-solubilizing agents in pesticide formulations could reduce the amount of organic solvent, producing eco-friendly pesticides for a safer environment.

### 3.7. Structural Changes in the Morphology of F. graminearum Mycelia Induced by GO, Fungicides and GO–Fungicides

**Figure 6:**
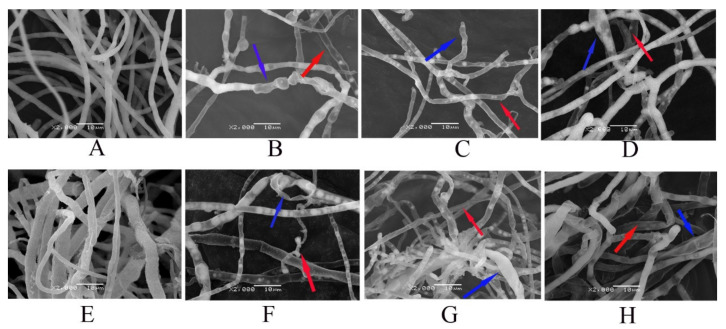
SEM images of the structural changes in the morphology of *F. graminearum* induced by fungicides, GO–fungicides and GO. Mycelia of the control (**A**), GO–treated (**E**, 100 µg/mL^−1^), Man–treated (**B**, 20 µg mL^−1^), Man–GO–treated (**F**, 20 µg mL^−1^), Cyp–treated (**C**, 100 µg mL^−1^), Cyp–GO–treated (**G**, 100 µg mL^−1^), Dif–treated (**D**, 200 µg mL^−1^) and Dif–GO–treated (**H**, 200 µg mL^−1^) *F. graminearum*, respectively. The position marked by a red arrow indicates hollowness, and that marked by a blue arrow indicates swelling.

SEM observation of mycelia of *F. graminearum* exposed to GO, fungicides and GO–fungicides revealed great differences in mycelial morphology. As shown in Figure 6A, the mycelia of normal *F. graminearum* displayed a typical lengthened, regular and homogeneous morphology with constant diameters and smooth external surfaces. After treatment with GO (Figure 6E), the mycelia were shrunk and had uneven thickness. However, the mycelia of *F. graminearum* became hollow, swollen and deformed after fungicide (Figure 6B–D) and GO–fungicide (Figure 6F–H) treatments. In Man and GO–Man treated *F. graminearum*, the mycelia were hollow, and many evident ovoidal- or spherical-shaped swellings were localized at either the subterminal positions of the apex or the middle positions along the mycelia. In the Cyp, Cyp–GO, Dif and Dif–GO treated *F. graminearum*, the middle or tip of the mycelia became swelled and hollow. The irregular swelling, thinning of cell walls and extensive hollowness observed in SEM are similar to those caused by other azole fungicides and chitosan [39,40].

Previous studies have suggested that mycelial morphological changes such as swelling are the results of nutritional and chemical stress, which are also associated with chemical shifts of cell wall composition [40,41]. It has been reported that the inhibitory effect of azole fungicides on ergosterol can affect the content of cell wall chitin, finally resulting in morphological changes of *Candida albicans*, such as swelling [40]. Yang and co-workers showed that the enhancement of chitinase and β-1,3-glucanase activity led to the increase of the hydrolysis of chitin and glucan, thus damaging the cell membranes of *pestalotiopsis theae* mycelia [28]. Moreover, another study demonstrated that an increase of chitin content in mycelial cell wall would result in morphological and ultrastructural changes of two chitosan-mediated wood-inhabiting fungi [41]. Thus, we suspect that, when the proper balance of cell-wall constituents is disrupted by some factors, such as fungicides and GO–fungicides, the pattern of cell-wall deposition and structure may be altered, such as ball-shaped cell-wall vesicles or swelled mycelia.

Furthermore, thin mycelial cell walls and hollow mycelia were found in all treatments with fungicides and GO–fungicides. In order to determine whether the internal structure of mycelial cells was changed, the morphology and cell wall texture of mycelia were further characterized by TEM.

**Figure 7:**
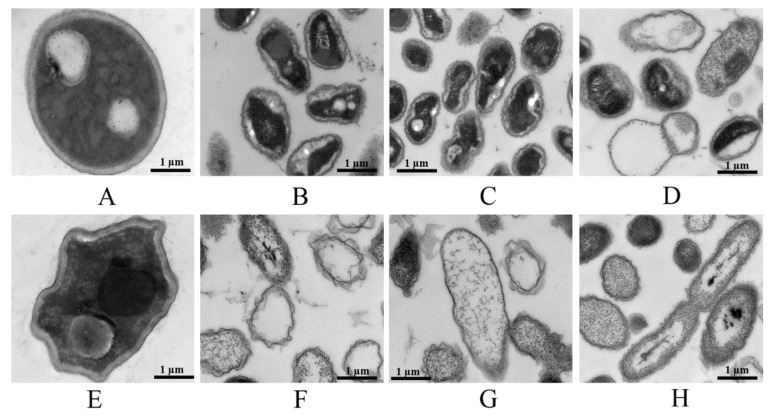
TEM images of structural changes in the morphology of *F. graminearum* induced by fungicides, GO–fungicides and GO. Mycelia of the control (**A**), GO–treated (**E**, 100 µg mL^−1^), Man–treated (**B**, 20 µg mL^−1^), Man–GO–treated (**F**, 20 µg mL^−1^), Cyp–treated (**C**, 100 µg mL^−1^), Cyp–GO–treated (**G**, 100 µg mL^−1^), Dif–treated (**D**, 200 µg mL^−1^) and Dif–GO–treated (**H**, 200 µg mL^−1^) *F. graminearum*, respectively.

TEM examination indicated that fungicides, GO–fungicides and GO induced ultrastructural changes in *F. graminearum*. As shown in Figure 7A, untreated mycelia contained dense cytoplasm and well-defined cell wall, and the integrity of membrane structure was maintained. The GO-treated mycelia were shrunk and deformed (Figure 7E). However, fungicide (Figure 7B–D) and GO–fungicide treatment (Figure 7F–H) brought about more significant changes in cellular structure in terms of both cell wall and cytoplasm. Particularly, the GO–fungicide treatment almost led to the disappearance of cytoplasm. Moreover, membrane fusion can be observed, which may be due to the changes in cell-membrane permeability and loss of cell-wall integrity caused by GO–fungicide treatment that resulted in cytoplasmic exosmosis. These results are consistent with the observed the damage of the cell-wall structure in the *P. thea* mycelia induced by Man fungicide [28].

The Man could lead to the dysfunction of mitochondria, depletion of cellular antioxidant enzymes and activation of the apoptotic pathways [42]. Although the antifungal mechanism of Man has no direct association with the cell wall, some ovoidal swellings can appear and rupture, resulting in a loss of cell-wall parts and extravasation of cytoplasm [43]. Cyp and Dif are membrane-specific azole fungicides, whose antifungal mechanism is to affect the sterol biosynthesis and then disrupt the function of fungal cell wall and cell membrane [44]. In addition, a previous study of our group has shown that GO can decrease lipid proteins and enhance histidine metabolism involved in cell-wall synthesis of the mycelia of *F. graminearum*, which could affect mycelial cell-wall synthesis and further affect the permeability of the cell membrane [45]. When GO is combined with Man, Cyp and Dif, their effects on cell wall and cell membrane are synergized. Thus, GO–fungicide treatment was found to have a more significant destructive effect on the cell wall and cell membrane of mycelia.

For chemical plant-disease management, various combinations of contact and systemic fungicides with different antifungal mechanisms have been proposed for the utilization of their synergistic effects, so as to broaden their spectrum of activity and delay the emergence of resistant fungal strains [46]. In the present study, GO and fungicides were found to have synergistic inhibitory effects on the spore germination, mycelial biomass and mycelial growth of *F. graminearum*. It has been reported that GO treatment can inhibit *F. graminearum* mainly by enriching the metabolic pathway and histidine metabolism. Moreover, the amount of lipid proteins involved in cell-wall synthesis would be decreased at the protein level, which would affect mycelial cell-wall synthesis [45]. Thus, the combination of GO with existing fungicides to formulate nano-fungicides may develop novel antimicrobial agents and may be a highly promising approach for disease control in agricultural science.

## 4. Conclusions

The present study provides evidence that the combination of GO with fungicides can bring about a synergistic inhibitory effect on *F. graminearum* in vitro and in vivo, with the magnitude of synergy depending on the ratio of GO and fungicide in the composite. At the optimal combination ratio, the fungicide adsorbed on the surface of GO can achieve the highest adsorption capacity and dispersibility. GO and fungicides can synergistically exert their antifungal activity when combined at appropriate ratios, which may help to significantly reduce the effective dose of antifungal agents. However, further studies are still needed to clarify the mechanism for the synergistic effect between GO and fungicides.

## Figures and Tables

**Table 1:** Treatments with single Man, Cyp and Dif or in combination with GO against *F. graminearum*.

Treatment	Concentrations (µg mL^−1^)
Control	acetone, tween 80 and water solution (AT) (1:1:98, v/v)
Man/Man–GO	2.5, 5, 10, 20
Cyp/Cyp–GO Dif/Dif–GO	12.5, 25, 50, 100 20, 40, 80, 200

**Table 2:** EC_50_ values of fungicides and GO–fungicides for inhibiting the mycelial growth, mycelial biomass and spore germination of *F. graminearum*.

Treatment	Slope ± SE ^a^	EC_50_ (μg mL^−1^) (95% CL) ^b^	SR ^c^
Man ^G^	2.69 ± 0.40	15.70(9.52~25.89)	—
Man–GO ^G^	3.40 ±0.28	7.80(5.98~10.17)	2.01
Cyp ^G^	2.52 ±0.75	40.65(24.75~63.19)	—
Cyp–GO ^G^	2.05+0.70	27.05(19.50~38.76)	1.50
Dif ^G^	4.37±0.15	5.80(3.57~9.43)	—
Dif–GO ^G^	4.69±0.21	2.33(0.95~5.72)	2.49
Man ^B^	3.66 ± 0.16	6.61 (5.61~7.80)	—
Man–GO ^B^	3.79 ± 0.31	4.09 (2.67~6.06)	1.61
Cyp ^B^	1.94 ± 0.25	40.94 (35.99~46.56)	—
Cyp–GO ^B^	2.69 ± 0.24	21.03 (17.99~24.60)	1.95
Dif ^B^ Dif–GO ^B^	4.29 ± 0.13 4.58 ± 0.11	17.83 (12.53~25.37) 7.12 (4.02–12.59)	— 2.50
Man ^S^	3.75 ± 0.14	14.20 (11.10~18.16)	—
Man–GO ^S^	4.16 ± 0.06	5.90 (5.39~6.46)	2.40
Cyp ^S^	3.45 ± 0.18	30.19 (22.97~39.67)	—
Cyp–GO ^S^	3.63 ± 0.06	17.16 (15.30~19.24)	1.76
Dif ^S^	3.44 ± 0.50	56.03 (51.05~61.47)	—
Dif–GO ^S^	3.89 ± 0.07	18.04 (14.41~22.58)	3.10

^a^ Slope of the probit mortality line. ^b^ EC_50_ values and the data in brackets are 95%confidence limits (CL). ^c^ Synergism ratio at EC_50_ values. ^G^ Mycelial growth test. ^B^ Mycelial biomass test. ^S^ Spore germination test.

**Table 3:** Control efficiencies of GO, fungicides and GO–fungicides on *F. graminearum* in the field.

Treatment (250 µg mL^−1^)	Disease Incidence (%) (7 d)	Disease Severity (%) (7 d)	Control Efficacy (%)
CK	81 ± 3.41 a	50.15 ± 1.21 a	—
GO	45 ± 1.91 b	33.42 ± 0.88 b	33.59 a
Man	43 ± 3.41 b	28.45 ± 0.90 c	43.26 b
Man–GO	21 ± 2.52 d	19.50 ± 0.93 e	61.19 d
Cyp	37 ± 3.00 bc	19.98 ± 0.56 e	60.17 d
Cyp–GO	19 ± 1.91 d	12.41 ± 0.36 f	75.26 e
Dif	32 ± 1.63 c	32.74 ± 0.58 b	34.72 a
Dif–GO	19 ± 1.91 d	24.58 ± 0.94 d	50.99 c

Different lowercase letters in Table indicate significant differences between treatments (*p* < 0.05).

## Data Availability

The data are included in the main text.
